# A New Approach to Quantify Semiochemical Effects on Insects Based on Energy Landscapes

**DOI:** 10.1371/journal.pone.0106276

**Published:** 2014-08-29

**Authors:** Rory P. Wilson, Rebecca Richards, Angharad Hartnell, Andrew J. King, Justyna Piasecka, Yogendra K. Gaihre, Tariq Butt

**Affiliations:** Biosciences, College of Science, Swansea University, Singleton Park, Swansea, Wales, United Kingdom; Natural Resources Canada, Canada

## Abstract

**Introduction:**

Our ability to document insect preference for semiochemicals is pivotal in pest control as these agents can improve monitoring and be deployed within integrated pest management programmes for more efficacious control of pest species. However, methods used to date have drawbacks that limit their utility. We present and test a new concept for determining insect motivation to move towards, or away from, semiochemicals by noting direction and speed of movement as animals work against a defined energy landscape (environmentally dependent variation in the cost of transport) requiring different powers to negotiate. We conducted trials with the pine weevils *Hylobius abietis* and peach-potato aphids *Myzus persicae* exposed to various attractants and repellents and placed so that they either moved up defined slopes against gravity or had to travel over variously rough surfaces.

**Results:**

Linear Mixed Models demonstrated clear reductions in travel speed by insects moving along increasingly energetically taxing energy landscapes but also that responses varied according to different semiochemicals, thus highlighting the value of energy landscapes as a new concept to help measure insect motivation to access or avoid different attractants or repellents across individuals.

**Conclusions:**

New sensitive, detailed indicators of insect motivation derived from this approach should prove important in pest control across the world.

## Introduction

Many insect species are implicated in a suite of problems ranging from acting as vectors of human and animal diseases [1], [2] to reducing crop yield [3], [4] that have significant socio-economic consequences worldwide [5]. The control of these pests is a complex issue that, in its infancy, generally relied on using large quantities of pesticides but which rapidly manifest a series of unwanted associated consequences [6], [7], [8]. Current attempts to minimize the use of inappropriate chemicals increasingly seek to define methods to attract insects so that pesticides and other mechanisms of control [9] can be applied to restricted areas and thus reduce potential ecosystem damage [10]. The most sophisticated approach of this type purports the use of a “push-pull” strategy which may, for example, use a combination of repellents and attractants to rarefy and concentrate insect densities in prescribed areas [11], [12], [13]. Pivotal in this, however, is the capacity to define the value of chemicals as attractants or repellents. There are two primary methods used for this; the first method places insects in a Y-tube olfactometer and exposes them to a chemical from one arm and a control from another [14]. The proportion of animals that move into each arm indicates the value of the substance as an attractant. The response per individual is, therefore, effectively binary (but see [15]). The second complementary method based on an electroantennogram (EAG), examines the voltage generated from the insect’s antennae as a result of exposure to odours, and measures the neurological degree of excitation in relation to exposure to chemicals [16], [17], [18]. This method requires appreciable expertise, equipment and time, does not help define the behavioural role of the chemical, may not necessarily allow for predictions with respect to insect behaviour under more natural conditions [19] and may not indicate whether an observed response will actually lead to movement in the field [20].

Studies on human preference typically use questionnaires whereby subjects are asked to quantify the degree to which they like or dislike something [21]. This has profound consequences for marketing because specific strategies can be directed at the variously reacting groups [22]. In non-human studies, motivational tests – as a measure of what animals want – have been developed and refined for some decades [23] and behavioural biologists interested in motivation from a welfare perspective have adopted a “consumer demand approach”. This tests an individual’s strength of preferences for a variety of potential resources, and classically involves animals working to push a weight-loaded door (with varying cost) in order to have access to the resources. This push-door paradigm was first used in hens by Duncan and Kite [24], and since then, adapted push-door paradigms have been used in a variety of species and contexts; most recently to test for cichlid fishes motivation for social partners and food [25]. Development of appropriate methodology to measure ‘what animals want’ has therefore enabled us to quantify motivation vertebrate preference for target resources, and provided a framework in which we can begin to assess animal emotion accurately [26], an important goal in animal welfare science [27].

We sought to create a system whereby insect motivation to move toward an attractant or away from a repellent could be precisely quantified so that all the advantages of similar approaches used in human studies and other vertebrates could be brought to bear to insect control. We capitalized on the recently proposed concept of ‘energy landscapes’, that a landscape can be defined in terms of the cost of travel (the energy used per unit distance) for an animal to move over it [28], [29]. We used two insect species as a proof of concept, the pine weevil, *Hylobius abietis*, and the peach potato aphid, *Myzus persicae*. In addition, for the pine weevil, we first tested it’s preference for different semiochemicals using a traditional Y-tube olfactometer, so that we could evaluate the benefit of our new approach. We created landscapes that could be changed to elicit variable movement costs before letting insects move over the surface, documenting their speed of movement as a function of both the attractant/repellent used and the difficulty of moving over the landscape. The speed of the study animals is a graded response to the prevailing conditions and thus should provide a measure equivalent to those stemming from human questionnaire preference studies, with all the benefits that this brings in designing insect control measures.

## Materials and Methods

### The Y-Tube Olfactometer

Experiments were conducted using a 90° 24 mm internal diameter, Y-glass tube olfactometer, model numbers OLFM-YT-2425F, OLFM-2425M and OLFM-IN-2425M from Analytical Research Systems, Inc., USA. The external diameter of the tube was 32 mm and arm lengths were 150 mm (long arm) and 85 mm (short arms). The Y-tube olfactometer was placed in a blacked out box to remove visual distractions.

### The Energy Landscape Olfactometer (ELO)

**Figure 1:**
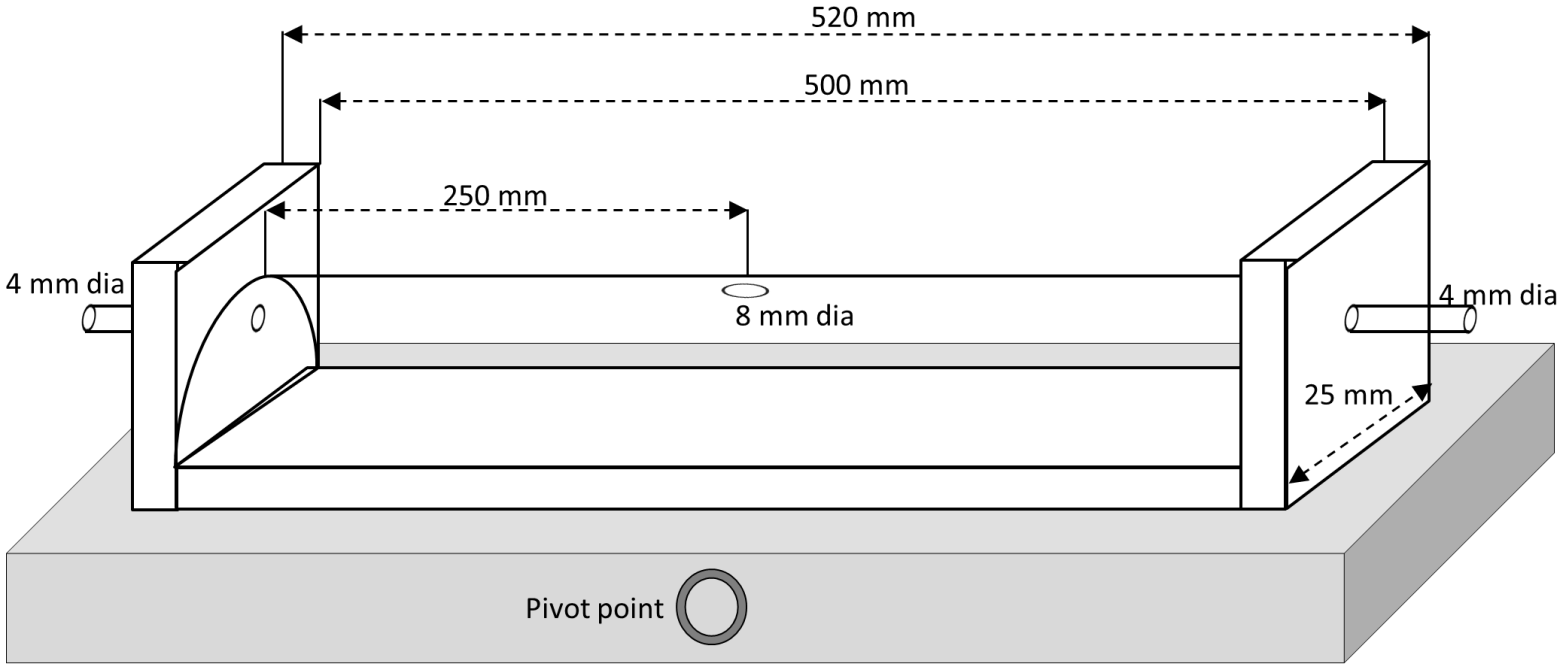
Schematic diagram of the incline energy landscape olfactometer showing the major features and dimensions. Note that the apparatus rests on a board (coloured grey) and that the angle of this board can be varied by rotation about a pivot point.

We constructed two energy landscapes Both landscapes were housed within a semi-circular high density Perspex tubing with closed ends (50 cm long×2.5 cm width×1.25 cm height) fixed to a flat 10 mm thick wooden base that was painted black to avoid visual stimuli [30]. At each flat end of the tubing 4 mm diameter tubes were inserted to allow for the introduction of an air flow with a chemical mix to be pushed along the tube at a defined rate. The semi-circular tubing had a central dorsal hole through which insects could be introduced and was marked at regular intervals so that insect speed could be noted (Fig. 1).

The ELO could be manipulated in two ways. The first involved tilting the landscape so that study insects approaching, or moving away from, the impinging chemical had to move up or down a known gradient, thus performing a known amount of work within a specified time to give a work rate metric. This was achieved by shifting the wooden base with pegs at known angles. The second manipulation was a change to the base floor, which was covered with Whatman filter paper. This paper could be variously roughened by sandpapers so that the fibres making up the matrix of the filter paper stood up, emulating trichomes with rough or smooth surfaces. Both systems could be combined. Olfactometers were thoroughly cleaned using appropriate solvents to ensure removal of chemical residues between assays between trials and filter papers renewed.

### Test insects and ethics statement

Two distinct insect species were considered as test examples for the energy landscapes, the pine weevil, *Hylobius abietis* Linneaus (Coleoptera: Curculionidae), and the peach potato aphid, *Myzus persicae* Sulzer (Hemiptera: Aphididae), representing very different types of animal. Preliminary tests showed that the weevils were virtually insensitive to the simulated trichome density but highly sensitive to movement gradient whereas the reverse was true of the aphids. Work on the two different species thus concentrated on using them in the energy landscapes to which they were most sensitive. Fresh insects were used in each assay. No permits were required for the described study, which complied with all relevant regulations.

### Semiochemicals and chemical reagents used

We used known attractant/repellent semiochemicals for the weevils and aphids at concentrations appropriate to elicit greatest activity. Test chemicals and solvents (Table 1) were purchased from Sigma Aldrich (UK) with the exception of garlic metabolic solution (GMS), seaweed extract and rapeseed oil, which were provided by Neem Biotech Ltd (UK).

**Table 1:** Test chemicals used in the Y-tube and energy landscape olfactometry studies.

Test Chemical	Solvent	Purity
α-pinene	Ethanol	98%
(−)-β-pinene	Ethanol	99%
3-carene	Ethanol	90%
Ethanol	n/a	95.0+%
Methanol	n/a	99.8+%
Hexane	n/a	99.8+%
α-terpineol	Hexane	≥96
(+)-citronella	Hexane	90%
Geraniol	Hexane	98%
Linalool	Hexane	96%
(+)-carvone	Hexane	96%
2-heptanone	Water	
Garlic Metabolic solution (GMS)	Water	100%
Seaweed extract	Hexane	low
Rape Seed Oil	Hexane	100%

For the pine weevils, we conducted basic Y-Tube olfactometer preference tests to ensure appropriate controls were used in our ELO experiments. Ethanol was used as a solvent due to the synergising effect it has with attractants such as α-pinene [31]. Ethanol was used as a control. Water was used to dilute the repellent garlic metabolic solution (GMS) and was also used as a control. All testing was undertaken using a 1% solution of either α-pinene, β-pinene, 3-carene and GMS.

For aphids, first, the repellency of different solvents (water, methanol, methanol+water, ethanol and hexane) was established. The most repellent solvent, hexane, was then used to dilute and enhance compounds known to have repellent properties. Hexane was used as a control. The exception was 2-heptanone which was dissolved in water in which case water was used as a control. Assays were performed using a 0.1% solution of the test compounds which included α-terpineol, (+)-citronella, geraniol, linalool, (+)-carvone, 2-heptanone, GMS, seaweed extract and rape seed oil. Aphids were attracted to Chinese cabbage, therefore, a Chinese cabbage leaf disc (4 cm diameter) was used as a positive (attractant) control.

### The Y-Tube Olfactometer (weevils)

Each arm of the Y-tube olfactometer was connected to an air flow of 0.3 L/min [32] after passing through black carbon filter. Filter paper (4×2 cm) was placed in each of the olfactometer arms with one filter paper being treated with 10 µl of the test solution (1%) and the other with 10 µl of the control solvent (i.e. ethanol or water). The solutions were pipetted onto the centre of the filter paper [33]. The test solutions were replaced every 2 days due to the volatility of the monoterpenes [34]. Twelve pine weevils were tested individually in each trial and trials were repeated three times. Each pine weevil was allowed 2 minutes to acclimatise to the new surroundings and then given 10 minutes to respond and walk towards either the known attractant or the control. Their response was recorded when the pine weevil had travelled at least 4 cm up the chosen arm and remained there for more than 10 seconds [33]. All experiments were conducted in daylight at 28°C (±2°C) and 60% relative humidity.

### Energy Landscape Olfactometer trials

Twelve pine weevils and 10 aphids were tested individually in each trial and trials were repeated three times. Animals were placed through the hole in the centre of the ELO and allowed to choose their travel direction. If the test animals moved toward the control or did not move after a period of 2 minutes, the behaviour was recorded and the animals removed and replaced. If the animals moved in the correct direction, travelling speed (mm/sec) towards the semiochemical at different inclines was recorded. No time was recorded in the 0–5 cm section to allow an ‘acclimatisation’ segment. The walking speed of individual animals was recorded (in mm/sec) at the 0° incline between 5 cm and 10 cm before the travel speed was then recorded for randomly chosen inclines (from one of four categories; 30°, 50°, 70° and 90°) for next three 5 cm sections. If the animal stopped or turned during this procedure, the behaviour was recorded, the animal removed and the apparatus re-cleaned.

A similar procedure was adopted for the aphids walking within the energy landscape olfactometer except that, instead of only tilting the apparatus to change the energy landscape, the aphids were also obliged to walk on either smooth or roughened filter paper. The latter was prepared by gently passing sandpaper over the surface four times in two alternating perpendicular directions. New filter paper was used for every trial.

Data stemming from this work were deposited in the Swansea University College of Science T-drive.

### Statistical analysis

Two-tailed binomial tests (SPSS 19, IBM Corp, 2010) were used to test for weevil preferences among semiochemicals in the Y-tube olfactometer. These tests assume a null hypothesis of no preference, and are commonly applied to Y-tube olfactometer analysis [35], [36].

To test the effect of chemical type, and landscape type, upon the speed (mm/s) with which the insects moved towards chemicals attractants and away from repellents, we ran two Linear Mixed Models (LMM) for each species, implemented in MLwiN (v. 2.25, 2011, Bristol University Centre for Multilevel Modelling, Bristol, U.K.). The weevil speed data did not follow a normal distribution and so was log_10_ transformed. In each model, trial number and date were fitted as random effects to control for potential non-independence across trials and within days. For our weevil dataset we fitted incline (continuous), and chemical attractant (categorical: see Table 1 for chemicals) as fixed effects. For the aphid dataset, we fitted incline (continuous), and chemical repellent (categorical: see Table 1 for chemicals) as fixed effects. We additionally fitted surface type (categorical: smooth, rough), and tested for an interaction between surface type and chemical.

Once we had determined the independent effects of chemical type, and energy landscape (incline, or surface type) in the above models, we ran two further models (one for each species) in which we allowed the effect of ‘incline’ to vary according to ‘chemical type’ (i.e. a random intercept, random slope model). This allowed us to see whether the insects would move faster (or slower) up steeper inclines according to the semiochemical present. This is similar to fitting multiple regressions of speed against incline; one for each chemical type, or fitting an interaction between chemical type and incline in our LMM, but is easier to interpret.

## Results

### Y-Tube Olfactometer

**Figure 2:**
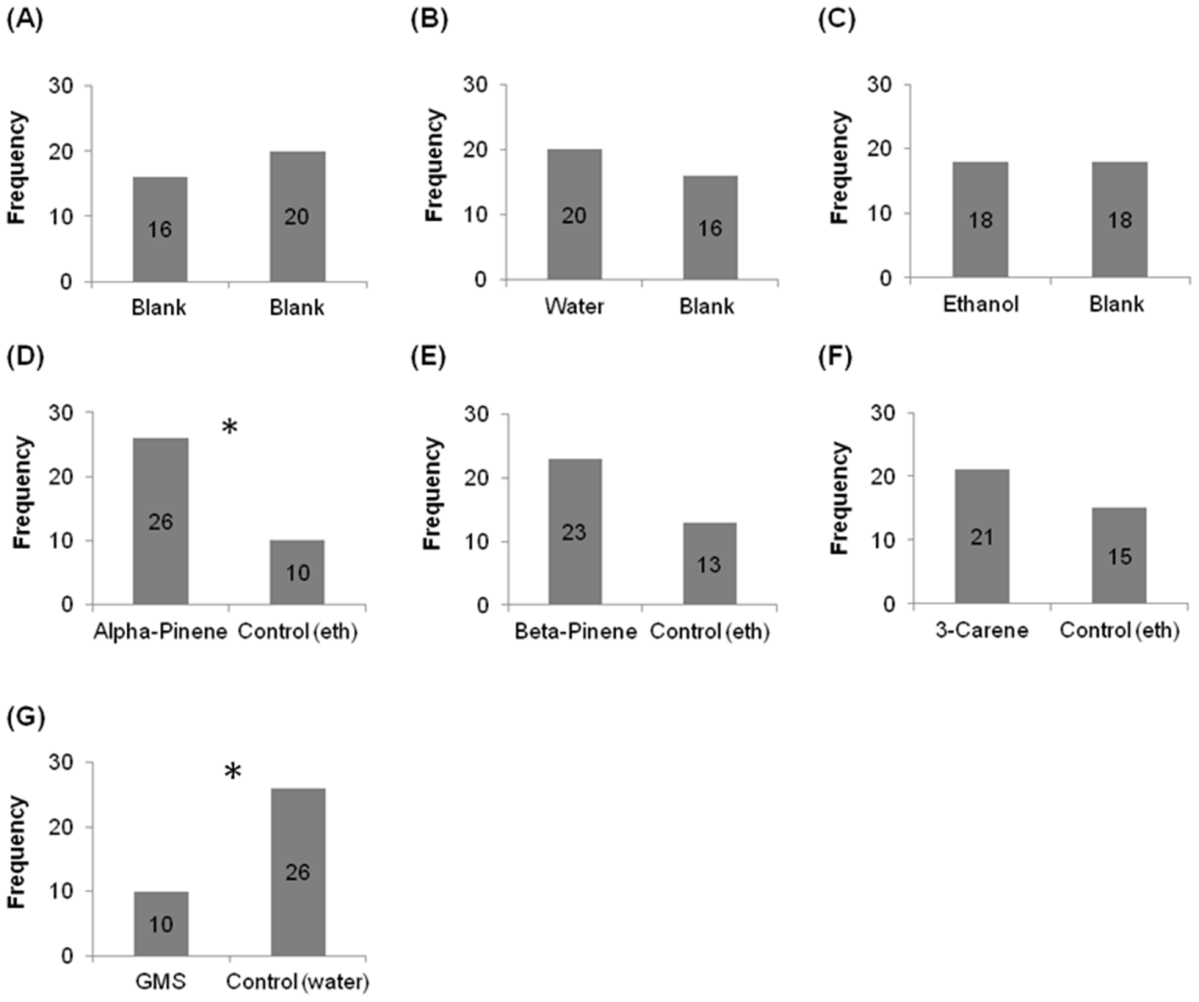
Y-tube olfactometer results for numbers of weevils choosing to walk towards either the semiochemical or the control for; (A) control [for examining arm preferences], (B) water [for examining differences between our control in A and water], (C) ethanol [for examining differences between our control in A and ethanol], (D) α-pinene, (E) β-pinene, (F) 3-carene and (G) garlic metabolic solution [GMS], all against the control for their respective solvents. Significant differences are indicated by (*).

Y- tube olfactometer tests showed weevils had no preference for our blank or control treatments (Figure 2A, 2B, 2C), and revealed an apparent preference for α-pinene, β-pinene, and 3-carene treatments, being apparently repelled by garlic metabolic solution. However, only α-pinene and garlic metabolic solution were statistically significant (Fig. 2D, 2E, 2F, 2G). We therefore investigated the preferences of the weevils to the semiochemical to which they were attracted, namely α-pinene, in our energy landscape olfactometer experiments.

### Energy landscape olfactometer

**Figure 3:**
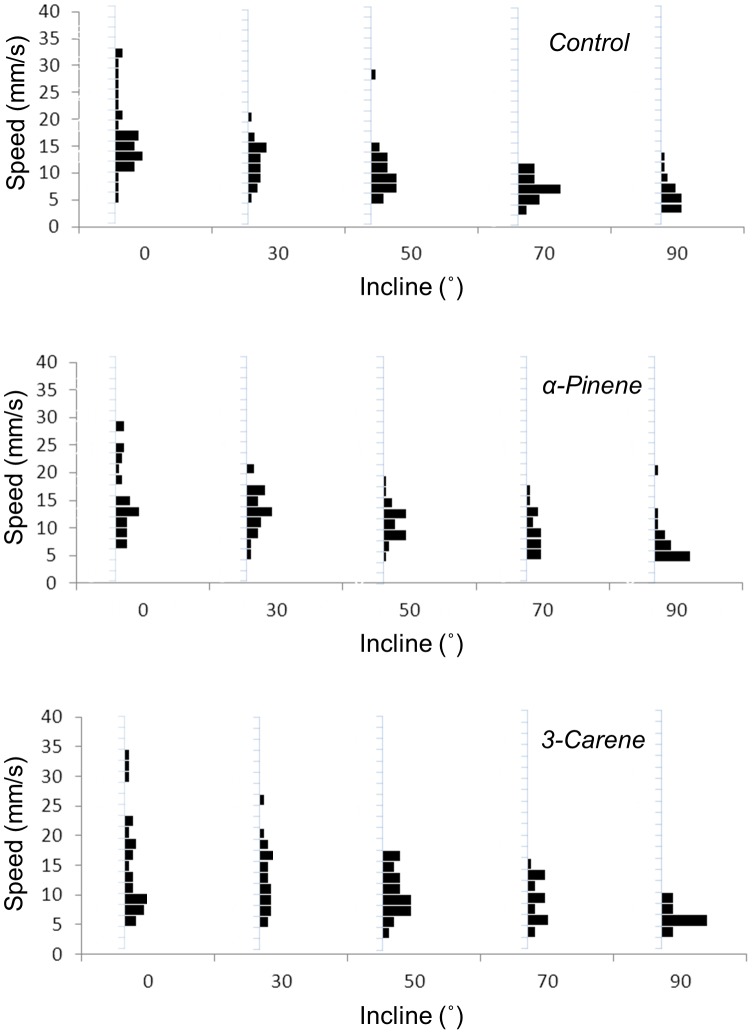
Examples of frequency histograms (expressed as a percentage of the total number of trials conducted for that condition) of the speeds at which pine weevils walked up specified inclines in response to selected semio-chemicals. Each condition consisted of 36 trials for weevils (see text).

**Figure 4:**
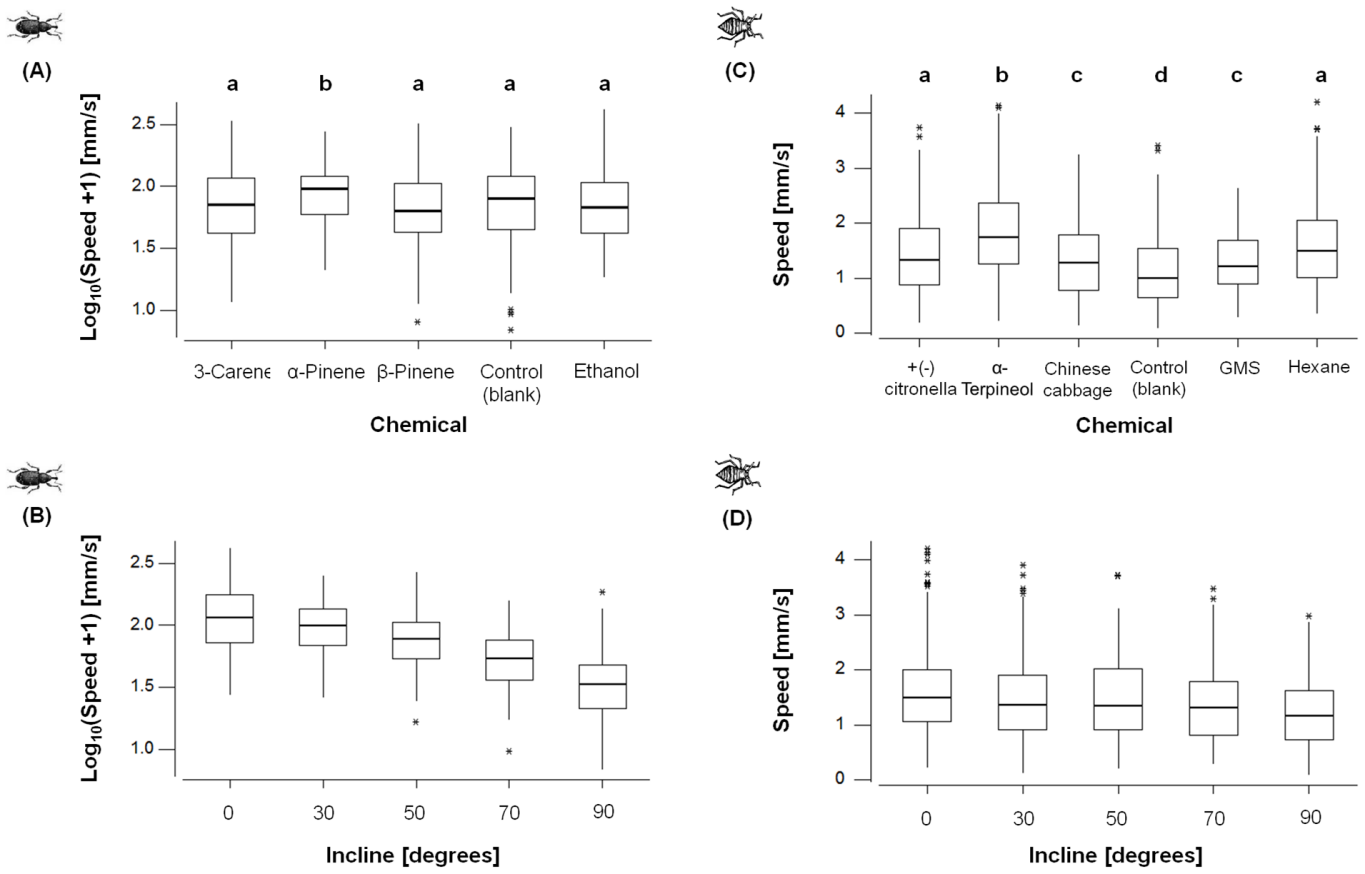
Speed at which aphids and weevils responded to different chemicals and the manner in which this changes with incline, represented by box and whisker plots indicating median (bold line), inter-quartile ranges (box), 95% confidence intervals (whiskers), and outliers (*). (**A**) Speed with which weevils move towards different chemical attractants (the control shown is the blank - Fig. 2a). The type of chemicals to which weevils were exposed significantly altered subjects approach speed (LMM: Wald = 10.86, df = 4, P = 0.028), with subjects moving faster towards α-pinene than all other treatments (significant pairwise differences (P<0.05) across chemical trials are indicated by different letters). (**B**) Speed with which weevils moved at different inclines (LMM: Wald = 75.21, df = 1, P<0.001). (**C**) Speed with which aphids move away from different chemical repellents (the control shown is the blank cf. Fig. 2a), which significantly affected aphid speed (LMM: Wald = 105.09 df = 5, P<0.001). Significant pairwise differences (P<0.05) across chemical trials are indicated by different letters. (**D**) Speed with which aphids move at different inclines(LMM: Wald = 75.21, df = 1, P<0.001). See methods for more details of models.

**Figure 5:**
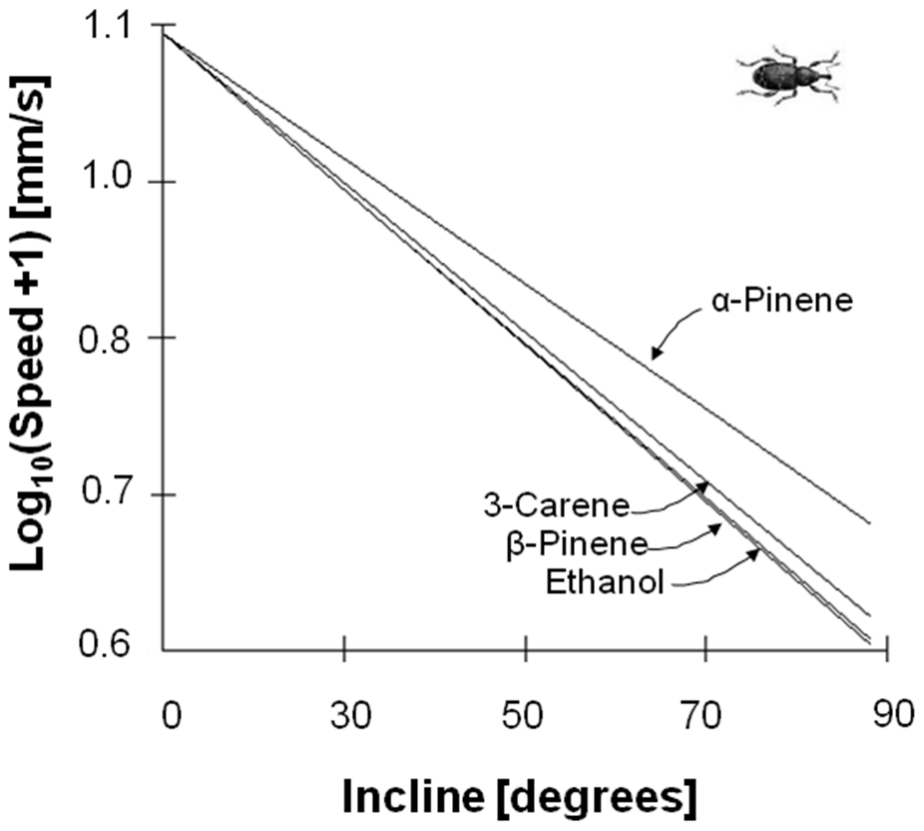
Model results for how incline is predicted to affect weevil speed. In this model, the intercept and slope of the effect of incline were allowed to vary with respect to chemical (i.e. a random slope, random intercept model) and our control condition was used as the reference category. See methods for further details.

Both insect species reacted to the energy landscapes by walking towards, or away from, the selected semiochemicals at speeds that showed considerable variation (Fig. 3). However, the type of chemicals to which weevils were exposed significantly altered subjects approach speed (Fig. 4a). The incline to which subjects were exposed also had an independent effect upon approach speed (Fig. 4b), and the magnitude of this effect differed with respect to chemical trial with, specifically, speed being maintained during α-pinene trials at steeper inclines than for other chemicals (Fig. 5).

**Figure 6:**
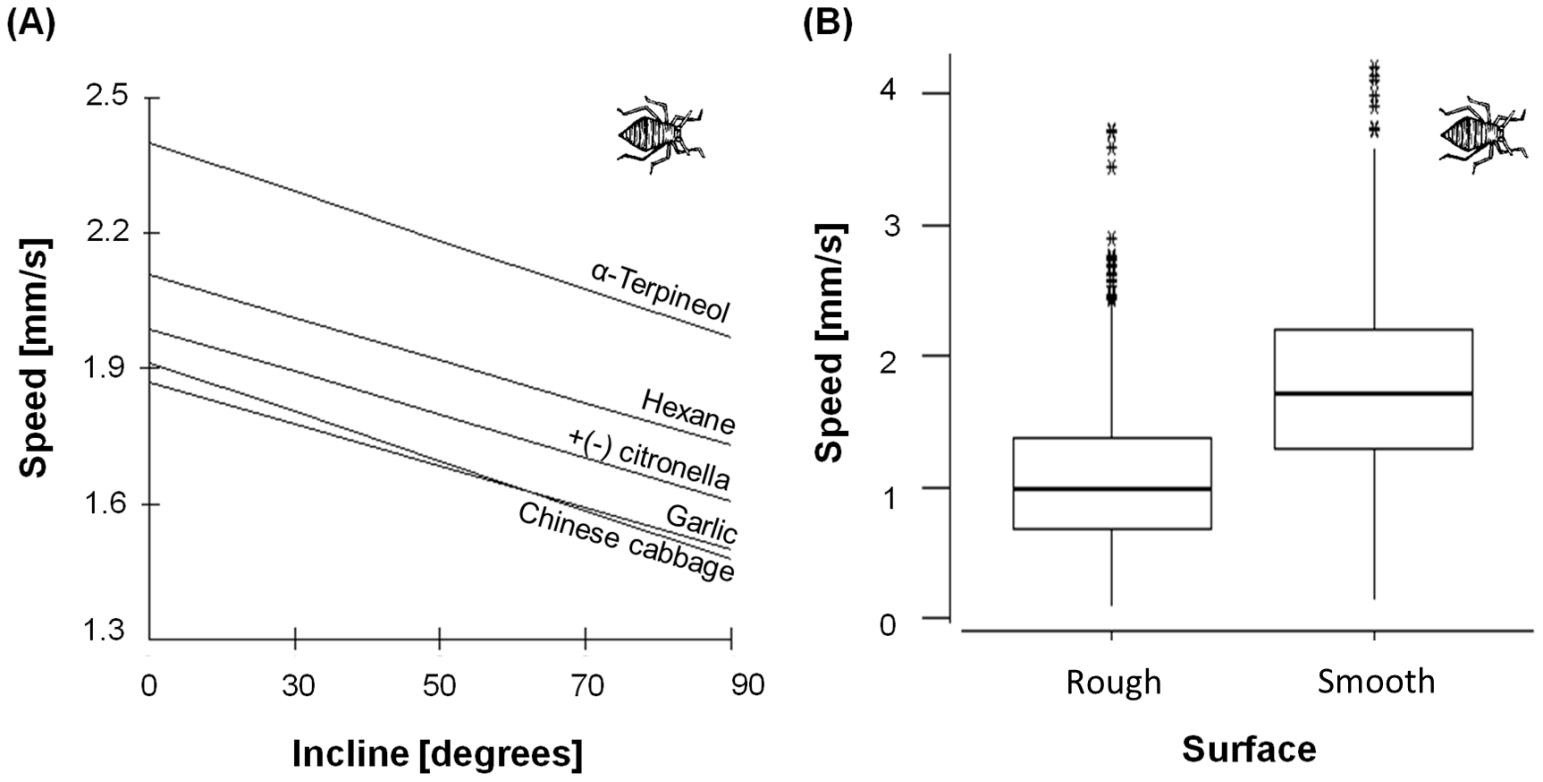
Changes in aphid movement speed as a function of incline and surface roughness. (**A**) The predicted effect of incline upon aphid speed to move away from chemicals, from a model in which the intercept and slope of the effect of incline is allowed to vary with respect to chemical (i.e. a random slope, random intercept model), and in which our control condition is used as the reference category. See methods for further details. (**B**) Box and whisker plot showing the overall effect of surface type upon speed (LMM: Wald = 93.94, df = 1, P<0.001); shown is the median (bold line), inter-quartile ranges (box), 95% confidence intervals (whiskers), and outliers (*).

The type of chemicals to which aphids were exposed also significantly altered travel speed (Fig. 4c), although they moved faster away from chemicals on flatter surfaces (Fig. 4d). However, the effect of incline on the speed with which subjects moved away was consistent across chemical repellents, with aphid speed compared to the control condition being fastest away from α terpineol and slowest away from Chinese cabbage (α terpineol>hexane>citrollena>garlic>Chinese cabbage: Fig. 6a). We also found that the aphids moved significantly slower away from chemicals on the rougher surface (Fig. 6b), and this effect differed across chemicals (LMM: Wald = 21.15, df = 5, P<0.001), with subjects moving quickest on the rougher surface when moving away from hexane compared to our control and all other chemicals (pairwise comparisons: P<0.01). Reductions in speeds on rough surfaces for other chemicals were not significantly different from one another (pairwise comparisons: P>0.05 in call cases).

## Discussion

The energy landscape olfactometer, using a prescribed energy landscape to assess insect motivation to move towards, or away from, stimuli such as semiochemicals, reveals a number of features in insect response that are not accessible using conventional olfactometers or antennograms. Although the new system effectively mirrors the conventional Y-tube olfactometer in offering one of two travel directions (or the choice to remain stationary), it also goes beyond the individual binary response of the Y-tube olfactometer by revealing motivation manifest in choice of speed. Although increasing the cost associated with different energy landscapes systematically reduced the speed of travel with any given semiochemical (Figs. 3–6), closer inspection revealed two more specific responses displayed to different semiochemicals. In the one case, there was minimal variation in speed of travel for pine weevils moving towards the different semiochemicals on the flat surface (Fig. 5) which would imply that there is apparently either no difference in the attraction of the different semiochemicals or that speed appears to be a poor measure of the attractiveness of semiochemicals. However, weevils responded to increased inclines by changing speed differentially according to semiochemical (Fig. 5). Conversely, aphids did show differences in their speed of movement away from the different semiochemicals at an incline of 0° (Fig. 6) but had decreases in speed with increasing incline that did not vary between semiochemicals with slope (Fig. 6a). Beyond this, however, aphids exhibited variation in speed according to whether the surfaces they travelled over were rough or smooth (Fig. 6b).

**Figure 7:**
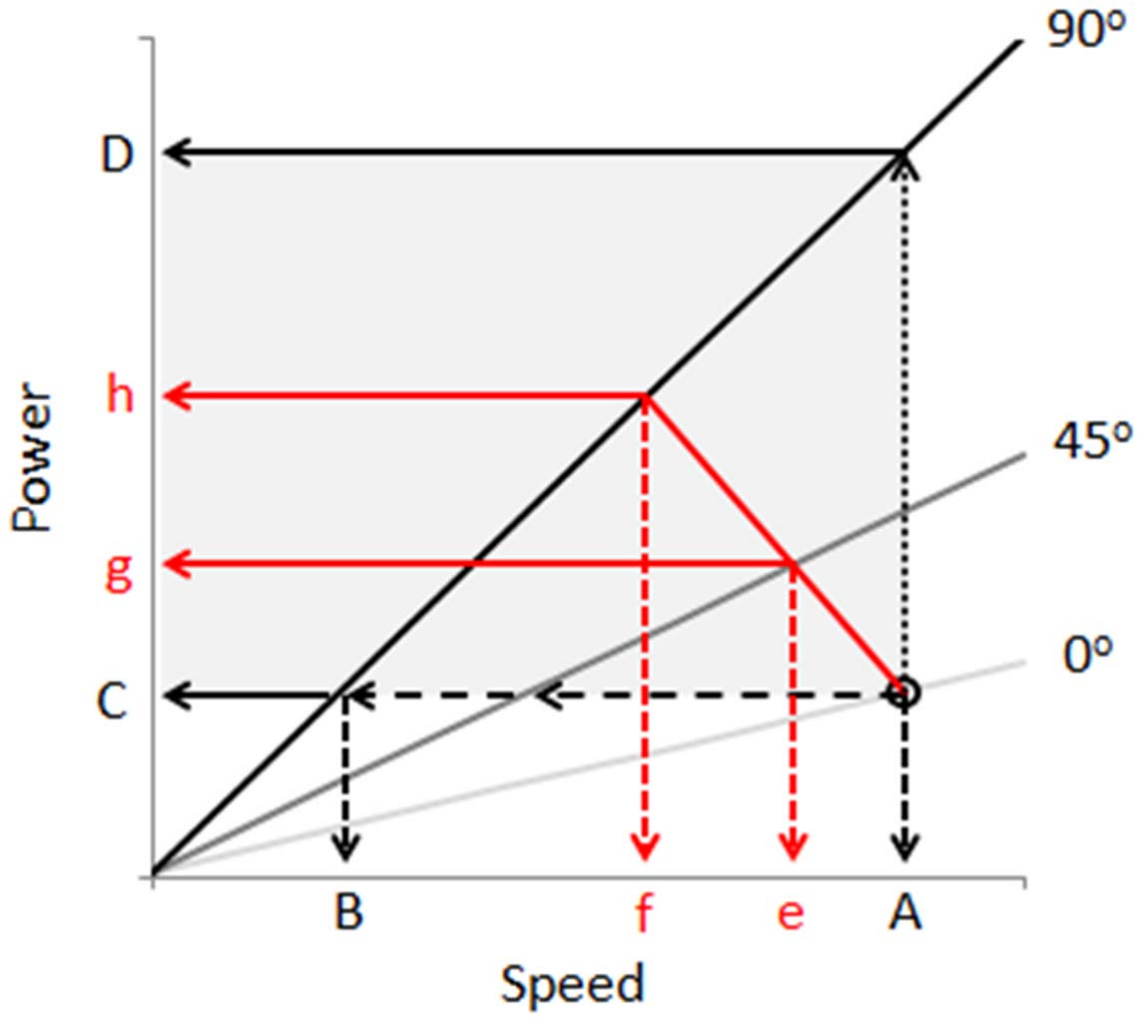
Schematic relationship between speed, incline and power of an insect climbing up 3 slopes of different inclines (after Full & Tullis 1990), showing how the results gained within the energy landscape olfactometer relate to energy expenditure. The grey box shows the proposed operational area of subjects. Walking towards an attractive semio-chemical, insects can either maintain power use at a constant level for the varying inclines, in which case speed is expected to drop with increasing incline (arrows terminating at A and B), or they can maintain speed, in which case power requirements increase with increasing incline (arrows terminating at C and D). In reality (red lines), animals are likely to operate somewhere between these two extremes (see arrows terminating in ‘e’ and ‘f’ for speed and ‘g’ and ‘h’ for power) with the more motivated subjects tending to maintain speed and incur increased power use with increasing incline.

In essence, speed indicates motivation because it affects the power costs selected by insects for movement towards, or away from, a defined goal. The more energy animals are prepared to expend per unit time to move towards or away from something, the stronger their motivational state is assumed to be. Full and Tullis [37] show how, in American cockroaches *Periplaneta americana* Linneaus, the rate of energy expenditure increases linearly with both speed and incline above resting metabolic rate (Fig. 7), a feature that seems common in both vertebrates and invertebrates [38]. We can use the general principles relating to the costs of terrestrial locomotion [38] to set up a framework within which to consider how insect motivational state may be reflected in the speeds and associated power costs that they choose for movement.

Faced with increasingly energetically onerous landscapes, such as increasing hair density or incline, insects may respond with one of two extreme responses or something in-between. They can either maintain their speed, which will lead to substantial increases in the rate of energy expenditure or they can maintain a constant power output, which will necessitate substantial decreases in speed. The response of our study animals seemed to be somewhere between these two extremes with, importantly, animals changing the way they respond according to the semiochemical used (Fig. 7). The fact that insects clearly do vary their elected power costs of travel according to energy landscape and stimulus, highlights an important difference between conventional Y-tube olfactometers, where incline is always 0°, and energy landscape olfactometers. This difference is apparent in comparison of the percentage positive responses in a Y-tube olfactometer to different semiochemicals (Fig. 2) with the slope of the regression of speed versus incline (Fig. 5) for the same semiochemicals derived by using energy landscape olfactometers. In pine weevils for example, the correlation coefficient for this is r^2^ = 0.53 (percentage data arcsin transformed to ensure normalization), which shows some concurrence, but the lack of a tight correlation presumably indicates the extent to which the ELO documents a different response; specifically, with the form of the speed/power change with incline (or hair density) with a given semiochemical, providing important information about motivational state in addition to that given by the conventional approach.

We propose, for our purposes, that an insect’s response to a semiochemical may be described hierarchically: The first, most coarse, level is whether the animal moves towards (or away from) the stimulus at all (and here we note that conventional olfactometer approaches do involve a loosely defined element of speed since subjects are required to have moved within a defined period of time). A second level specifically measures that movement speed while a third, and final, level measures how that speed relates to defined, energetically variable movement constraints. Indeed, we could convert our measured speed values with respect to incline and/or trichome density to power costs using respirometry experiments similar to those conducted by Full and Tullis [37], [39] on cockroaches. Pragmatically however, the simple measurement of speed should be enough to indicate motivation, especially given that the relationship between speed and metabolic rate is so prescribed [38].

The value in effectively measuring the work rates exhibited by insects in response to semiochemicals is most obvious in ‘push-pull’ operations [11], designed to attract pests to specific sites where they can be treated with pesticides. Insects operating in the wild will be exposed to natural variation in their own energy landscapes via incline, surface roughness, trichome density, wind strength, surface roughness etc., so a demonstration of metabolically costly movement across landscapes is a powerful indicator of semiochemical value. Beyond consideration of the overall response however, the frequency distribution of speeds used under the varying energy landscapes (Fig. 3) also highlights whether there are differentially motivated groups within the sample taken [40]. Bimodality in travel speed, for example, may indicate a difference in susceptibility to semiochemicals between e.g. sexes or nutritional state [41], something that may prove important in control measures. This issue would have been better defined in our own experimental protocol if we had followed marked individuals through various energy landscapes. Future work can address this, and perhaps clarify how individuals vary over time.

An apparent inconvenience in using inclines as energy landscapes for pine weevils, and a number of other species, is the preference for these insects to walk up inclines anyway (Fig. 3). We effectively dealt with this tendency by having controls, and cognisance of this will be important for trials on any species that displays a similar reaction to inclines. Our controls allow speed as a function of incline to be compared directly to that exhibited during a semiochemical experiment but future work may prefer to simply subtract semio-chemical-induced mean speeds from those of controls although this simpler approach may obscure some patterns. However, experimental protocol with proper controls can deal with this so that the effect can be subtracted from their response. A climbing pattern was not obvious in our aphid experiment. In fact, incline is much less important in modulating travel speed in aphids than in pine weevils. In essence, the work done in climbing should be related to the gain in potential energy (E_p_), given by E_p_ = mgh, where m is the mass, g is the gravitational constant and h is the height climbed, so mass-specific work should be the same for both species, with the rate of work given by the speed and the incline. However, smaller animals have higher mass-specific resting metabolic rates while travel costs scale linearly with mass [39], [42], which explains why Lipp et al. [43] found no obvious difference in VO_2_ *versus* incline in ants. Indeed, the effect of an elevated resting metabolic rate with respect to travel costs means that anything that slows down rates of travel increases the costs of travel correspondingly and it is for this reason that we designed the hair-density energy landscape as an additional experimental protocol for the aphids. The concept also elicited the expected result (Fig. 6b).

Our work presents two types of energy landscape olfactometers as case studies to assess their utility in insect pest programs. The results are very encouraging, demonstrating that the way insects modulate their speed in relation to the difficulty of traversing variable terrain provides a more complex, and certainly different, response to conventional methods using Y-tube olfactometers or EAGs. Thus, the approach gives both another useful measure of semiochemical attraction or repellency and highlights, and gives metrics for, a more intricate response pattern. Future studies could examine energy landscapes produced in a variety of others ways such as using air currents or a variably constructed ‘vegetation’-simulating matrix for flying insects or differential surface textures and substrates for walking or burrowing species, respectively. Such work should help verify the real value of energy landscape olfactometers as an additional tool to help our attempts to control pests with minimum detriment to the environment.
